# Pulmonary vascular adaptation to high-altitude living

**DOI:** 10.1186/2050-6511-14-S1-O9

**Published:** 2013-08-29

**Authors:** Martin Wilkins

**Affiliations:** 1 Imperial College London, South Kensington Campus, London SW7 2AZ, UK

## 

When a rat is exposed to a low oxygen (10%) atmosphere, pulmonary artery pressure rises over a few days to reach a new level around 2 to 3-fold above that in a normal oxygen atmosphere. The rise in pulmonary artery pressure is accompanied by structural changes in the pulmonary vasculature that include the expansion of the smooth muscle in pulmonary arteries and arterioles and extension to previously unmuscularised vessels. The right ventricle undergoes hypertrophy.

This response of the pulmonary circulation to hypoxia is typical of most mammals, including humans. There is, however, considerable inter-species and within species variation in the magnitude of the response. For example, Tibetans, who have lived at altitude for many thousands of years, exhibit a lower pulmonary artery pressure at altitude than more recent arrivals, such as the Han Chinese.

Recognition of this differential response has prompted studies comparing the activity of candidates, including components of nitric oxide signalling pathway, in cohorts of subjects at high altitude. Access to high-throughput sequencing has enabled studies comparing genome sequences in these cohorts. Understanding the genomic variants that underlie susceptibility to hypoxia-induced pulmonary hypertension can signpost druggable signalling pathways that might be exploited to ameliorate the effects of exposure to hypoxia – and may also be useful in diseases where hypoxia is a pathological factor (such as COPD) or in intensive care situations.

